# Exploring the stability of the gender gap in faculty perceptions of gender climate at a rural regional university

**DOI:** 10.1371/journal.pone.0301285

**Published:** 2024-04-02

**Authors:** Michael J. Bordieri, Paula J. Waddill, Qiaofeng Zhang, Maeve L. McCarthy, Claire Fuller, David Balthrop

**Affiliations:** 1 Department of Psychology, Murray State University, Murray, KY, United States of America; 2 Department of Earth and Environmental Sciences, Murray State University, Murray, KY, United States of America; 3 Department of Mathematics and Statistics, Murray State University, Murray, KY, United States of America; 4 Department of Biological Sciences, Murray State University, Murray, KY, United States of America; 5 Department of Global Languages and Theatre Arts, Murray State University, Murray, KY, United States of America; Babes-Bolyai University, Cluj-Napoca, ROMANIA

## Abstract

Increasing awareness of gender barriers and biases in academic institutions is an essential component of institutional change strategies to promote equity and inclusion. There is an established perception gap in recognizing gender inequities in the workplace, whereby men faculty under acknowledge the stressors, barriers, and biases faced by their women faculty colleagues. This study explored the gender gap in faculty perceptions of institutional diversity climate at a rural comprehensive regional university in the United States. In addition to gender, differences across academic discipline and time were explored using 2 (men and women) x 2 (STEM and other) x 2 (2017 and 2022) between-groups ANOVAs. Results revealed a gender gap that persisted across time and perceptions of stressors, diversity climate, student behavior, leadership, and fairness in promotion/tenure procedures, with marginalized (women) faculty consistently reporting greater barriers/concern for women faculty relative to the perceptions of their men faculty colleagues. These findings are largely consistent with the extant literature and are discussed both with regard to future research directions and recommendations for reducing the perception gap and addressing institutional barriers to gender equity.

## Introduction

Gender is a social construct that consists of characteristic norms, roles, and behaviors associated with gender categories, such as women and men [1]. Gender is also hierarchical [1], and there is well-documented historical and contemporary evidence of gender inequity in professional occupational contexts, whereby women are both underrepresented and underpaid relative to men, both generally [2–5] and within academia, specifically [6–8]. Gender differences in the perception of these and other gender biases are also well established in both the early [9, 10] and contemporary literature [11, 12, as reviewed by 13]. This perception gap appears to persist across professional contexts and workplaces including medical residents [14], otolaryngologists [15], and corporate managers [16]. Of primary relevance to the current investigation, García-González et al. [17] recently explored perceptions of gender bias in academic research institutions across Spain and found that men were less likely than women to perceive gender bias in their academic workplace; this difference persisted across country, research area, position, and type of institution.

The gender perception gap presents a significant obstacle to addressing systemic inequities and fostering institutional change. In addition to perpetuating misunderstanding and minimizing of the experiences of underrepresented faculty, the perception gap might also undermine the development and implementation of effective policies that address these disparities. Privilege has long been theorized to be invisible to those who possess it [18], and the impact of privilege in shaping diversity in higher education has been subject to detailed review [19, 20]. Upper-level university administrators remain disproportionately men [21] and thus may be more likely to downplay or fail to recognize the barriers faced by women faculty, including subtle discrimination. Likewise, underrepresented faculty may feel discouraged from voicing experiences and concerns that do not align with the “rosy” climate perceptions held by their majority-identified colleagues and leaders, thereby further minimizing their experiences and limiting the identification of climate barriers. A better understanding of the gender perception gap among faculty in higher education could increase awareness of climate barriers faced by women faculty and help inform efforts to close the gap.

Over the past 20 years campus climate surveys have become a valuable tool for both institutional leaders and equity researchers interested in better understanding the structural and cultural climate barriers faced by underrepresented faculty members in higher education [22–24]. Broadly defined, campus diversity climate surveys aim to elicit feedback from science, technology, engineering, and mathematics (STEM; S1 Table) faculty and often non-STEM faculty regarding their perceptions of opportunities, barriers, stressors, and other indicators of workplace climate that impact career satisfaction, advancement, and retention. Work-life balance/conflicts [25, 26] are often a core focus of climate surveys although institutions often customize surveys to focus on a range of phenomena, including microaggressions [25], faculty workloads [27], and fit/inclusion [28]. Many of these climate surveys are conducted as part of larger institutional change efforts funded by the National Science Foundation (NSF) ADVANCE program, which is designed to increase the representation and career success of women in STEM disciplines [29].

The purpose of the current study was to explore gaps in the perception of campus diversity climate among faculty at a rural comprehensive regional university in Kentucky. Faculty were surveyed on their perceptions of stressors, general department climate for women, students’ behavior towards women faculty, gender equity in leadership/influence, and gender equity in promotion/tenure policies across two time points (2017 and 2022). The addition of a second time point five years after the initial climate survey allowed for the direct exploration of the stability of gaps in perception over time, which to our knowledge has yet to be directly explored in the literature. We hypothesized that the well-established gap between majority identified (i.e., men) and underrepresented (i.e., women) faculty would be present across both climate domain and time point. We also explored whether our hypothesized gender perception gap interacted with academic discipline (STEM vs. non-STEM).

## Method

### Surveys

Our variables of interest were drawn from a climate survey we developed by adapting (with permission) items from climate surveys administered under the auspices of ADVANCE programs at Oakland University [30], University of California—San Diego [31], University of North Texas [32], Washington University in St. Louis [33], and Western Washington University [34] as well as additional items that we created ourselves. This study was reviewed and approved by the Murray State University Institutional Review Board (IRB) for the protection of human subjects (S1 File; IRB# 16–098; S2 File; &IRB# 20–001; S3 File). After viewing an initial page with informed consent information that included a description of the participant’s role in the study and notification of approval of the research by the Murray State University IRB, participants provided informed consent by clicking “Continue.” The survey asked questions on a variety of themes including university and department climate, workload, work/family balance, mentoring and networking, and tenure and promotion as well as demographic items. The initial edition of the survey consisted of 71 items and was administered electronically in the spring of 2017 (February 15^th^ to March 15^th^) by the Survey Research Institute (SRI) at Cornell University. The second edition of the survey consisted of 64 items that included many of the same items from the first edition as well as some new ones; it was administered electronically in the spring of 2022 (February 28^th^ to March 18^th^) by SRI. Participants responded to items by rating their perceptions on 4- or 5-point Likert scales.

#### Sample and data

In both 2017 and 2022, participants were recruited via an invitation email with a link to the survey that was sent by SRI to all full-time faculty. The 2017 survey was sent to 519 faculty, and 373 faculty provided survey responses (a response rate of 72%). The 2022 survey was sent to 484 faculty, and 264 faculty responded (a response rate of 55%). Chi-square goodness of fit tests indicated that the distribution of category frequencies for gender, rank, and race/ethnicity of the survey respondent samples did not differ significantly from those of the original population of all faculty to whom the survey was sent (for 2017, smallest *p* = .906; for 2022, smallest *p* = .17). Table 1 presents participant demographics for both surveys.

**Table 1:** Demographic characteristics of respondent samples.

	2017				2022			
	STEM		Non-STEM		STEM		Non-STEM	
	Women (%)	Men (%)	Women (%)	Men (%)	Women (%)	Men (%)	Women (%)	Men (%)
Total across all categories	35.3	64.7	58.5	41.5	46.5	43.5	61.8	38.2
Race/Ethnicity								
Asian	5.7	13.9	5.6	5.6	7	15.1	1	6.4
Black or African American	0	3	6.4	2.2	4.6	1.9	3	0
Hispanic or Latino/a	0	0	2.4	1.1	2.3	1.9	3	0
American Indian or Alaska Native	0	0	0	0	0	0	0	0
Native Hawaiian or Other Pacific Islander	1.9	0	0	0	0	0	0	0
White	92.4	83.1	85.6	91.1	86.1	81.1	92	92
Two or more races	0	0	0	0	0	0	1	1.6
Current Rank								
Instructor	34.5	12.9	22.8	23.3	30.4	17	20.6	17.5
Assistant Professor	36.4	31.7	38.6	26.7	30.4	22.6	35.3	19.1
Associate Professor	10.9	22.8	24.4	24.4	17.4	39.6	27.4	27
Professor	18.2	32.6	14.2	25.6	21.8	20.8	16.7	36.4

#### Independent variables

The independent variables in the current analysis were gender and discipline (STEM, non-STEM). The determination of a participant’s discipline as STEM was based upon Classification of Instructional Programs (CIP) codes that the U.S. Department of Homeland Security has designated as STEM disciplines [35] as well as disciplines related to the programs in the National Science Foundation’s Directorate for Social, Behavioral and Economic Sciences [36]. As a result, faculty were categorized as STEM if their primary responsibility lay in one of the following programs/departments: agricultural science, animal and equine science, biological sciences, chemistry, computer science and information systems, economics, earth and environmental sciences, engineering and physics, mathematics and statistics, occupational safety and health, political science and sociology, psychology, veterinary technology and pre-veterinary medicine.

#### Dependent variables

The dependent variables in the current analysis focused on a subset of items from the larger survey. These items assessed participants’ perceptions of several aspects of the workplace Table 2 presents the items constituting each variable.

**Table 2:** Survey items constituting each dependent variable.

Variable	Items
Overall stress (amount of stress)	• household responsibilities • childcare • caring for someone who is ill, disabled, aging or with special needs • meeting day-to-day work expectations • the way your personal life and work interfere with each other • having a successful academic career • your salary • subtle discrimination • the amount of support in your department/unit • the racial, ethnic or cultural climate at the university • time to do your research/scholarship/creative activities • the climate for women at the university • time to spend with your spouse/partner or significant other • opportunities to network with colleagues • time to spend with your family
General Climate (level of agreement)	• The climate for women faculty in my department/unit is good. • My department/unit has difficulty retaining women faculty. • Faculty in my department/unit are serious about treating men and women faculty equally • Generally speaking, women faculty in my department/unit must work harder than men to convince colleagues of their competence. • Women faculty in my department/unit who have young or school age children are considered to be less committed to their careers than women colleagues without children. • Faculty men in my department/unit who have young or school age children are considered to be less committed to their careers than colleagues who are men without children.
Student Behavior (level of agreement)	• Students at this university treat women faculty differently than men faculty. • Students at this university do not respect women faculty as much as men faculty.
Leadership/Influence (level of agreement)	• My department/unit has made an effort to promote women faculty into leadership positions. • Most faculty in my department would be would be as comfortable with a woman chair/director as a man chair/director. • Women faculty in my department/unit are less likely than their counterparts who are men to have influence in departmental/unit politics and administration. • Faculty men are more likely than faculty women to be involved with informal social networks within the department/unit.
Tenure/Promotion (level of agreement)	• When it comes to tenure decisions in my department/unit, criteria are applied to women faculty in the same way as to men faculty. • When it comes to promotion decisions in my department/unit, criteria are applied to women faculty in the same way as to men faculty

*Overall stress* was evaluated as an average score across 15 items for which participants rated the amount of stress they felt about each item using a 4-point scale (where 1 = *none*, 2 = *very little*, 3 = *some*, 4 = *a great deal*). *Perceived general department/unit climate for women* was evaluated with six items for which participants rated their agreement with each statement on a 5-point scale ranging from 1 = S*trongly disagree* to 5 = *Strongly agree*. *Perceptions of students’ behavior toward women faculty* was assessed with two items for which participants rated their level of agreement with each statement on a 4-point scale ranging from 1 = *Strongly disagree* to 4 = *Strongly agree*. Respondents also had the option to indicate “Do not know” for each student behavior item; however, *do not know* responses were not included in subsequent analyses of the student behavior variable. *Perceived leadership/influence* was measured with four statements for which participants rated their agreement with each on a 5-point scale that ranged from 1 = *Strongly disagree* to 5 = *Strongly disagree*. *Perceived equity in tenure and promotion* was assessed with two items that were each rated on a 5-point scale ranging from 1 = *Strongly disagree* to 5 = *Strongly agree*.

## Results

Principal components analyses of each set of items in each of the previously described dependent variables supported our conceptually derived grouping of survey items. Cronbach’s alpha was used to examine the internal reliability of the items for each aspect. Cronbach alpha values ranged from .75 to .94 and confirmed that the items within each aspect were closely related. To assess the role of gender and STEM discipline as well as any differences between 2017 and 2022 response patterns, separate 2 x 2 x 2 between-groups ANOVAs were performed for the items in each aspect with a family-wise significance level set at .05. Table 3 presents the means for the individual items in each group and the pattern of results is summarized in Table 4.

**Table 3:** Average ratings with standard deviations in parentheses.

	2017				2022			
	STEM		Non-STEM		STEM		Non-STEM	
	Women (*SD*)	Men (*SD*)	Women (*SD*)	Men (*SD*)	Women (*SD*)	Men (*SD*)	Women (*SD*)	Men (*SD*)
Overall stress^a^	2.80 (.51)	2.62 (.60)	2.76 (.58)	2.62 (.60)	2.88 (.58)	2.52 (.61)	2.77 (.61)	2.66 (.73)
General Climate								
Good climate for women	3.78 (1.17)	4.15 (.90)	4.15 (.90)	4.32 (1.72)	3.55 (1.04)	4.28 (.95)	3.78 (1.06)	4.05 (.87)
Difficulty retaining women	2.43 (1.10)	2.48 (1.21)	2.02 (.95)	1.88 (.81)	2.83 (1.08)	2.13 (1.15)	2.40 (1.00)	2.83 (1.18)
Serious about treating men and women equally	3.79 (1.03)	4.07 (1.05)	3.98 (1.10)	4.15 (1.10)	3.58 (1.10)	4.21 (.91)	3.78 (.99)	4.03 (1.06)
Women must work harder to be seen as competent	2.72 (1.42)	1.82 (1.12)	2.43 (1.38)	1.72 (1.04)	3.36 (1.28)	1.98 (1.23)	2.82 (1.42)	2.21 (1.23)
Women faculty with young children considered less committed	2.57 (1.25)	1.77 (1.04)	2.37 (1.30)	1.82 (1.12)	3.07 (1.28)	1.74 (1.08)	2.62 (1.32)	2.10 (1.14)
Men faculty with young children considered less committed	2.09 (.99)	1.95 (1.17)	1.93 (1.02)	1.91 (1.12)	2.00 (.99)	1.84 (1.05)	1.95 (.91)	2.15 (1.24)
Student Behavior								
Treat women faculty differently than men^a^	2.70 (1.08)	2.15 (1.06)	2.70 (.92)	2.47 (.92)	3.06 (.91)	2.14 (1.12)	2.92 (.86)	2.79 (1.07)
Respect women faculty less than men^a^	2.65 (1.07)	2.10 (1.02)	2.61 (.94)	2.38 (.95)	2.86 (.94)	2.00 (1.10)	2.81 (.92)	2.61 (1.10)
Leadership/Influence								
Most faculty as comfortable with woman department chair as man	3.57 (1.14)	4.08 (1.00)	4.12 (1.04)	4.42 (.98)	3.56 (1.24)	4.40 (.92)	3.95 (1.15)	4.28 (.92)
Women less likely to have department influence	2.36 (1.56)	1.97 (1.13)	2.08 (1.06)	1.64 (.97)	2.61 (1.26)	1.74 (.90)	2.57 (1.21)	1.98 (.98)
Effort made to promote women to leadership	3.24 (.99)	3.46 (.99)	3.78 (.98)	3.77 (.97)	3.50 (1.13)	3.96 (1.06)	3.87 (1.01)	4.12 (.86)
Men more likely to be involved in informal networks	2.74 (1.04)	2.15 (1.04)	2.56 (1.03)	2.20 (1.11)	2.91 (1.27)	2.08 (1.10)	2.62 (1.12)	2.18 (1.11)
Tenure/Promotion								
Tenure decision criteria applied the same way to women and men	3.72 (1.10)	4.39 (.84)	4.18 (1.00)	4.44 (.89)	3.46 (1.29)	4.66 (.66)	3.94 (1.9)	4.18 (1.03)
Promotion decision criteria applied the same way to women and men	3.88 (1.41)	4.55 (.80)	4.19 (1.23)	4.53 (.88)	3.40 (1.43)	4.62 (.90)	4.00 (1.43)	4.31 (1.17)

Ratings on a 5-point scale except where indicated.

^a^Rated on a 4-point scale

**Table 4:** Overall gender and STEM effects collapsed across year.

Item	Gender	STEM	Gender x STEM
Overall stress	Women > Men**	NS	NS
General Climate			
Good climate for women	Women < Men***	NS	NS
Difficulty retaining women	NS	STEM > NonSTEM***	NS
Serious about treating men and women equally	Women < Men***	NS	NS
Women must work harder to be seen as competent	Women > Men***	NS	NS
Women faculty with young children considered less committed	Women > Men***	NS	NS
Men faculty with young children considered less committed	NS	NS	NS
Student Behavior			
Treat women faculty differently than men	Women > Men***	NS	STEM***
			NonSTEM: NS
Respect women faculty less than men	Women > Men***	NS	STEM***
			NonSTEM: NS
Leadership/Influence			
Most faculty as comfortable with woman department chair as man	Women < Men***	NS	NS
Women less likely to have department influence	Women > Men***	NS	NS
Effort made to promote women to leadership position	Women < Men**	STEM < NonSTEM***	NS
Men more likely to be involved in informal networks	Women > Men***	NS	NS
Tenure/Promotion			
Tenure decision criteria applied the same way to women and men	Women < Men***	NS	STEM***
			NonSTEM: NS
Promotion decision criteria applied the same way to women and men	Women < Men***	NS	NS

Only items significant at the relevant Bonferroni-adjusted alpha level are indicated.

*p < .05.

**p < .01.

***p < .001.

### Overall stress

There were no significant main effects of year or STEM discipline nor were there any interactions of these factors with each other or with gender (all *F*s < 1). The only significant effect was the main effect of gender, *F*(1, 296) = 6.64, *MSE* = 82.50, *p* = .01. Women’s overall stress average across the 15 items (*M =* 2.78, *SD* = 0.58) was greater than men’s overall stress, *M =* 2.62 (*SD* = 0.62). The effect size (Cohen’s d) for this difference was .28.

### General climate

To control error rate across multiple testing across multiple items, a Bonferroni correction was applied based on the six ANOVAs (one for each item in this aspect), yielding a per-item significance threshold of .008. There was no significant main effect of year for any of the items nor did year interact with any other factor. Furthermore, with the exception of perceptions regarding difficulty in retaining women faculty, there were no significant main effects or interactions of STEM discipline. For that retention item, faculty in STEM disciplines perceived a significantly greater difficulty by their department in retaining women faculty (*M* = 2.46, *SD =* 1.16) than faculty in non-STEM disciplines (*M* = 2.13, *SD* = 1.02), *F*(1, 576) = 11.76, *MSE* = 1.4, *p* = .0006, Cohen’s d = .30. Although women in general had a greater tendency to perceive that their department had a difficult time retaining women faculty (*M* = 2.32, *SD* = 1.07) than did men faculty (*M* = 2.20, *SD* = 1.11), the main effect for gender did not reach the Bonferroni-adjusted significance threshold (*p* = .015). On the other hand, there was a significant main effect of gender in perceptions of the department climate for women, *F*(1, 578) = 21.67, *MSE* = 0.89, *p* < .0001, how serious colleagues are about treated women and men faculty equally, *F*(1, 593) = 13.67, *MSE* = 1.10, *p* = .0002, the extent to which women faculty have to work harder to be seen as competent, *F*(1, 606) = 66.92, *MSE* = 1.63, *p* < .0001, and the commitment of women faculty with young children, *F*(1, 605) = 59.31, *MSE* = 1.45, *p* < .0001.

Overall, women faculty were less likely than men to perceive the climate in their department as good for women (women: *M* = 3.88, *SD* = 1.04; men: *M* = 4.20, *SD* = 0.85; Cohen’s d = -.33). Women were also less likely than men to perceive that their department was serious about treating women and men faculty equally (women: *M* = 3.83, *SD* = 1.06; men: *M* = 4.11, *SD* = 1.04; Cohen’s d = -.27). They were more likely to feel that women had to work harder than men to convince colleagues of their competence (women: *M* = 2.72, *SD* = 1.42; men: *M* = 1.90, *SD* = 1.15; Cohen’s d = .64) and that women faculty with young children were seen as less committed to their careers than men with young children (women: *M* = 2.57, *SD* = 1.31; men: *M* = 1.85, *SD* = 1.09; Cohen’s d = .60). In contrast, was no significant main effect of gender (*F* < 1) or of any other factor nor were there significant interactions of any factors in regard to perceptions about the commitment of men faculty with young children (smallest *p* = .18).

### Student behavior

To control error rate, a Bonferroni correction was applied based on the two ANOVAs (one for each item in this aspect), yielding a per-item significance threshold of .025. There was no significant main effect of year nor did year interact with any other factor in the ratings of perceived student behavior toward women faculty. However, there was a significant main effect of gender that was modified by a significant interaction of gender with discipline for the perception that students treat women faculty differently than men faculty, *F*(1, 411) = 7.45, *MSE* = 0.95, *p* = .0066. Tests of simple effects indicated that a significant gender difference for STEM faculty, *F*(1, 411) = 21.73, *p* < .0001, but no difference for non-STEM faculty, *F*(1, 411) = 1.75, *p* = .1862. STEM women (*M* = 2,86, *SD* = 1.02) had a stronger perception than STEM men (*M =* 2.15, *SD =* 1.07) that students treat women faculty differently, but the difference between perception of non-STEM women (*M* = 2.79, *SD* = 0.90) and non-STEM men (*M* = 2.60, *SD* = 0.99) was not significant.

The same pattern of a significant interaction of gender and discipline held for the perception that students do not respect women faculty as much as men, *F*(1, 413) = 5.65, *MSE* = 0.98, *p* = .0178. Tests of simple effects indicated a significant gender difference for STEM faculty, *F*(1, 413) = 19.47, *p* < .0001, but no difference for non-STEM faculty, *F*(1, 413) = 2.60, *p* = .1073. STEM women (*M* = 2.74, *SD* = 1.01) had a stronger perception of less student respect of women than did STEM men (*M* = 2.07, *SD* = 1.04) whereas the difference between non-STEM women (*M* = 2.70, *SD* = 0.93) and non-STEM men (*M* = 2.47, *SD* = 1.01) was not significant.

### Leadership/Influence

To control error rate across multiple testing across multiple items, a Bonferroni correction was applied based on the four ANOVAs (one for each item in this aspect), yielding a per-item significance threshold of .0125. There was no significant main effect of year for any of the items nor did year interact with any other factor. Furthermore, with the exception of perceptions regarding efforts made to promote women to leadership positions, there were no significant main effects or interactions of STEM discipline. There was, however, a significant main effect of gender for all items. Overall, women faculty (*M* = 3.90, *SD* = 1.14) were significantly less confident than men faculty (*M* = 4.28, *SD* = 0.97) that most of the faculty in their department would be as comfortable with a woman being department chair as with a man, *F*(1, 592) = 29.99, *MSE* = 1.10, *p* < .0001, Cohen’s *d* = -.36. Compared to men (*M* = 1.84, *SD* = 1.03), women had a stronger perception (*M* = 2.36, *SD* = 1.17) that women faculty were less likely than men to have influence in their department, *F*(1, 589) = 36.33, *MSE* = 1.19, *p* < .0001, Cohen’s *d* = .47. Women (*M* = 2.66, *SD* = 1.09) had a stronger perception than men (*M* = 2.16, *SD* = 1.08) that faculty men were more likely than faculty women to be involved with informal department networks, *F*(1, 587) = 33.84, *MSE* = 1.18, *p* < .0001, Cohen’s *d* = .46, In terms of departmental efforts to promote women to leadership positions, although both women and men faculty agreed such efforts were being made, women overall had a lower perception (*M* = 3.68, *SD* = 1.03) than men (*M* = 3.77, *SD* = 1.00), *F*(1, 577) = 6.93, *MSE* = 0.99, *p* = .0087, Cohen’s *d* = -.10. In addition, STEM faculty in general (*M* = 3.52, *SD* = 1.05) perceived less effort to promote women into leadership in their departments than did non-STEM faculty (*M* = 3.86, *SD* = 0.97), *F*(1, 577) = 15.89, *p* < .0001, Cohen’s *d* = -.34.

### Tenure/Promotion equity

To control error rate, a Bonferroni correction was applied based on the two ANOVAs (one for each item in this aspect), yielding a per-item significance threshold of .025. Ratings for the tenure perception item were collected only from faculty who were tenured or on the tenure track. Ratings for the promotion perception item were collected only from faculty who had been promoted.

There was no significant main effect of year nor did year interact with any other factor in the ratings of either item. However, there was a significant main effect of gender that was modified by a significant interaction of gender with discipline for the perception that tenure criteria are applied equally to men and women, *F*(1, 502) = 12.15, *MSE* = 1.05, *p* = .0005. Tests of simple effects indicated a significant gender difference for STEM faculty *F*(1, 502) = 38.29, *p* < .0001, but the difference for non-STEM faculty did not reach the significance threshold, *F*(1, 502) = 4.44, *p* = .0356. STEM women (*M* = 3.61, *SD* = 1.18) were less confident than STEM men (*M =* 4.48, *SD =* 0.79) that tenure decision criteria were applied to women and men faculty in their department in the same way; however, non-STEM women (*M* = 4.07, *SD =* 1.14) and non-STEM men (*M* = 4.34, *SD* = 0.95) held more similar perceptions.

In terms of promotion criteria, there was no significant main effect of discipline nor did it interact with any other factor. However, there was a significant gender difference, *F*(1, 260) = 17.91, *MSE* = 1.32, *p* < .0001, Cohen’s d = -.47. Women (*M* = 3.96, *SD* = 1.36) were significantly less confident than men (*M* = 4.50, *SD* = 0.94) that promotion decision criteria were applied to women and men in their department in the same way.

## Discussion

This paper presents the results of two climate studies administered in 2017 and 2022 by the Murray State University ADVANCE team. The studies assessed the perception of gender equality at a regional comprehensive university in rural Kentucky, USA. Overall, there was strong evidence that men and women faculty in STEM and non-STEM disciplines experienced and perceived gender inequities differently, with men faculty consistently perceiving a stronger gender diversity climate than women faculty. This pattern of findings is consistent with the established literature on the perception gap in gender equity in the workplace [13–16]. Further, these findings extend the work of García-González and colleagues [17] by replicating the gender gap among faculty in a non-research-intensive institution in the United States.

Overall, women faculty were less likely than men to perceive the climate in their department as good for women. Furthermore, the extent to which women faculty have to work harder to be seen as competent and the commitment of women faculty with young children were greater issues of concern for women faculty. Women were also less likely than men to perceive that their department was serious about treating women and men faculty equally. Women were more likely to feel that women had to work harder than men to convince colleagues of their competence and women were more concerned that women faculty with young children were seen as less committed to their careers than men with young children. These findings provide further evidence of the gap between men and women faculty perceptions of gender diversity climate and highlight the “invisible” nature of privilege [18], with men faculty consistently perceiving a rosier climate for their women faculty colleagues than what their colleagues actually perceived.

Women faculty also reported greater perceived stress than men. The impact of this stress and broader climate concerns may have a cumulative negative effect that is overwhelming for women faculty [37, 38]. Further, the impact of stressors and climate may at least partially account for the lack of progress in the representation of women in STEM departments and leadership roles [39, 40]. The leaky pipeline continues to be impacted by these issues, and it may be a case of “injury by hundreds of little cuts.” Our findings support this possibility, as we observed relatively small but significant gaps for women faculty that could cumulatively have a significant negative effect on climate, retention, and advancement. Administrators may mistakenly view these concerns in isolation as small and insignificant. Further, administrators might also ignore these concerns because of the gap in perception observed in our findings. For example, they could adopt the faulty view that since the majority of faculty are content with the institutional climate, the overall climate is fine. Institutional change strategies that involve increased awareness and allyship among men faculty and administrators may be especially well-suited to target this perception gap [41, 42].

Faculty in STEM disciplines perceived greater difficulties in retaining women faculty. The lack of representation of women in STEM disciplines may be a contributing factor to this issue [43, 44]. STEM women were less confident than STEM men that tenure decision criteria were applied to women and men faculty in their department in the same way. Our analysis also indicated that STEM women perceived that students treat women faculty differently than men faculty, and that students do not respect women faculty as much as men faculty. These findings are consistent with well-established bodies of literature documenting gender biases in student evaluations of teaching [45, 46] and the promotion and tenure process [47, 48]. Women faculty were also less confident that faculty in their department would be as comfortable with a woman department chair as with a man. Additionally, women perceived that they were less likely to have influence in the department and that men were part of informal networks and STEM faculty perceived less effort being made to promote women to leadership positions than non-STEM faculty. These findings are consistent with broader literature regarding challenges faced by women in academic leadership roles [39, 40].

Our results show no significant effect of year, indicating that problems with perceptions have not substantially changed in the five years between surveys. This provides direct evidence of the stability of the gender perception gap within an institution. This finding is consistent with indirect evidence from literature that suggested stability in the effect over time across studies and samples [9–12]. While the stability of the effect is not surprising, it is important to consider the broader institutional context during this time frame, as we implemented an ADVANCE Adaptation grant between 2017 and 2022 with the goal of increasing awareness of gender equity and increasing instructional support for women faculty [49]. In this regard, the observed invariance across time could be seen as an indication that the gender climate did not improve as a result of the ADVANCE initiatives. However, it is important to note that the COVID-19 pandemic also occurred between our survey timepoints, and the pandemic has been linked to a clear increase in barriers and stressors for women faculty [50, 51]. Thus, the observed stability in climate could be seen as an indicator of the success of ADVANCE initiatives in protecting against the unequal impacts of the pandemic. Future research is needed to explore the unique impact of the COVID-19 pandemic on gender diversity climate as well as the potential for equity interventions to narrow the gender perception gap.

The obtained findings are not without limitations. While survey items were based on previous climate surveys and assessed for construct fit using principal components analysis and internal consistency, they lack formal psychometric validation. As this research area matures, future studies should seek to more rigorously validate climate measures by establishing more robust evidence of reliability and validity, including establishing predictive validity with faculty retention and advancement outcomes. Surveying faculty across two time points is a strength of the current study, as we found that the observed gender perception gap was largely invariant across time. However, due to confidentiality concerns during data collection, it was not possible for us to match faculty responses across time points. Thus, this study is not able to speak to how each participant’s perceptions may have changed over time, and future studies should consider collecting data in a way that allows for robust within-subject comparisons. Another limitation is that this study operationalized gender as a binary and did not assess the intersection of gender with other marginalized identities. In particular, this study measured gender identity using binary gender self-reported by faculty to human resources. Gender identity is fluid, especially among nonbinary individuals [52]. Future research should assess gender identity concurrent with other survey measures and use a more inclusive measure of identity, including non-binary, transgender, cis-gender, and self-description response options. In addition, research has established that women faculty of color [53], women faculty who identify as lesbian/bisexual and gender non-binary faculty [54], and women faculty with disabilities [55] face additional barriers and challenges. Future climate survey research should employ an intersectional lens to better contextualize the experiences of marginalized faculty.

While the obtained results provide clear evidence of a gender perception gap, they do not identify the cause(s). A recent review by Lee et al. [13] proposed social dominance theory [56] as a potential motivation for privileged groups (e.g., men) to downplay the discrimination experiences of members of minority groups. Additionally, Wu and Dunning [57, 58] have observed that members of majority groups (including men) display cognitive performance deficits in recognizing discrimination in the first place, so defensive motivations might only partially explain gender differences in the perception of bias. These recent studies highlight possible psychological mechanisms that maintain the gap in gender perceptions in academic environments, and they also support the need for interventions to specifically target and close this perception gap. Meaningful and lasting institutional change to support women and other unrepresented faculty in STEM and non-STEM disciplines requires a focus on transforming both institutional policies and climate [29, 59].

The US is projected to become more racially and ethnically diverse in the next decades, continuing the trajectory that started over half a century ago [60]. Higher education institutions are responsible for advancing the economic and social well-being of all [61], and they play a critical role in a functional pluralistic society [62]. The diversity of college campuses in which faculty, staff, and students come together to learn, teach, and grow amid varying viewpoints and perspectives is key to students’ academic and social growth [63]. This growth and the concomitant institutional satisfaction of all students leads to increased recruitment and retention of members of underrepresented groups [64]. In addition, a diverse faculty positively impacts graduation rates of not only underrepresented minority students but also students of all races/ethnicities [65].

Broadening participation in STEM is an important avenue toward meeting the needs of a more diverse and capable workforce [43, 44]. Low-income, first-generation, and under-represented minority students face significant barriers to attending and graduating from college, particularly in STEM fields [66–68]. The underrepresentation of women in STEM is well documented in the literature [69, 70]. This phenomenon has been attributed to factors like gender stereotypes, lack of social support networks, unwelcoming and sometimes hostile academic climate, and gender biases [7]. Furthermore, perceptions of sexism within the immediate academic environment are not only detrimental for women but are also associated with a higher sense of academic impostorism and lower self-efficacy and feeling of belonging, all of which could lead women doctoral students in STEM fields to drop out [71] and thus further reduce the diversity of the pool of future faculty. On the other hand, less bias can be related to better performance. Smeding [72] found that women engineering students held weaker implicit gender-STEM stereotypes compared to other groups and that those weaker biases were less negatively related to math grades.

Based on findings from 177 institutions that received NSF ADVANCE grants between 2001 and 2018, Casad [7] identified policies, interventions, and a positive organizational climate as effective approaches to increase the representation of women faculty in STEM fields. The current results add to this growing body of literature that is focused on a more comprehensive consideration of the experiences of women and other underrepresented faculty in STEM (see [73] for a review). Systemic efforts, including efforts funded by NSF ADVANCE programs [29], that target the improvement of campus climate and the gender perception gap highlighted in this study have the potential to further improve diversity in the STEM workforce.

In summary, findings from two climate surveys five years apart revealed a persistent perception gap between men and women faculty, particularly in STEM disciplines. Men faculty underestimated the challenges and stressors faced by their women faculty colleagues and overestimated positive indicators of gender diversity climate. These findings are broadly consistent with the existing literature on the gender perception gap, and this study replicated previous research in academic settings by extending the findings to faculty at a rural comprehensive regional institution. Further, this study provided direct evidence of the stability of the perception gap over a five-year interval. Future research is needed to explore the gender perception gap using more psychometrically sound measures that also include a broader intersectional focus on marginalized faculty identities beyond binary gender. Targeted interventions, such as programs that enhance awareness and allyship among men faculty, may help bridge this perception gap and foster increased support for broader institutional change strategies designed to enhance gender equity.

## Supporting information

S1 Table List of abbreviations. (DOCX)

S1 File PLOSOne human subjects research checklist. (DOCX)

S2 File Human subjects protocol I.D.–IRB # 16–098 approval letter. (PDF)

S3 File Human subjects protocol I.D.–IRB # 20‐001 approval letter. (PDF)
