# Supplementary material for: Exploring the stability of the gender gap in faculty perceptions of gender climate at a rural regional university
Source: PLoS One. 2024 Apr 2;19(4):e0301285. doi: 10.1371/journal.pone.0301285 (PMC10986963; doi:10.1371/journal.pone.0301285)
Supplement: S1 Table — (DOCX) [file pone.0301285.s001.docx]

S1 Table. List of Abbreviations.

| Abbreviation | Meaning |
| --- | --- |
| ANOVA | Analysis of Variance |
| CIP | Classification of Instructional Programs |
| COVID-19 | Coronavirus Disease 2019 |
| F | Ratio of explained variance / unexplained variance |
| IRB | Institutional Review Board |
| M | Mean |
| MSE | Mean Squared Error |
| NS | Non-(statistically) significant |
| NSF | National Science Foundation |
| p | Probably of obtaining the observed or more extreme effect given the assumption of no effect (null hypothesis) is true |
| SD | Standard Deviation |
| SRI | Survey Research Institute |
| STEM | Science, Technology, Engineering, and Mathematics |
