# Supplementary material for: Exploring the stability of the gender gap in faculty perceptions of gender climate at a rural regional university
Source: PLoS One. 2024 Apr 2;19(4):e0301285. doi: 10.1371/journal.pone.0301285 (PMC10986963; doi:10.1371/journal.pone.0301285)
Supplement: S2 File — (PDF) [file pone.0301285.s003.pdf]

MURRAY STATE UNIVERSITY

Institutional Review Board

328 Wells Hall
Murray, KY 42071-3318
270-809-2916● msu.irb@murraystate.edu

TO: Maeve McCarthy
Mathematics and Statistics

FROM: Institutional Review Board
Jonathan Baskin, IRB Coordinator

DATE: 12/7/2016

RE: Human Subjects Protocol I.D. – IRB # 16-098

On behalf of the MSU IRB, I have completed my review of your finalized participant consent page and survey for your Level 1 protocol entitled *Differences and Deficits Affecting Women STEM Faculty: Creating a Framework for Change at a Rural Public University.* I have determined that your research, as described in the protocol form, will be conducted in compliance with Murray State University guidelines for the protection of human participants.

The forms and materials that have been approved for use in this research study are attached to the email containing this letter. These are the forms and materials that must be presented to the subjects. Use of any process or forms other than those approved by the IRB will be considered misconduct in research as stated in the MSU IRB Procedures and Guidelines section 20.3.

This Level 1 approval is valid until 2/22/2017.

If data collection and analysis extends beyond this time period, the research project must be reviewed as a continuation project by the IRB prior to the end of the approval period, 2/22/2017. You must reapply for IRB approval by submitting a Project Update and Closure form (available at murraystate.edu/irb). You must allow ample time for IRB processing and decision prior to your expiration date, or your research must stop until such time that IRB approval is received. If the research project is completed by the end of the approval period, then a Project Update and Closure form must be submitted for IRB review so that your protocol may be closed. It is your responsibility to submit the appropriate paperwork in a timely manner.

The protocol is approved. You may begin data collection now.

Opportunity *afforded*

murraystate.edu

Equal education and employment opportunities M/F/D, AA employer. Murray State University supports a clean and healthy campus. Please refrain from personal tobacco use.

MURRAY STATE UNIVERSITY

Institutional Review Board

328 Wells Hall
Murray, KY 42071-3318
270-809-2916● msu.irb@murraystate.edu

TO: Maeve McCarthy
Mathematics and Statistics

FROM: Institutional Review Board
Jonathan Baskin, IRB Coordinator

DATE: 3/8/2017

RE: Amendment to Human Subjects Protocol I.D. – IRB # 16-098

The IRB has completed its review of the amendment submitted for your Level 1 protocol entitled *Differences and Deficits Affecting Women STEM Faculty: Creating a Framework for Change at a Rural Public University*. After review and consideration, the IRB has determined that the changes, as described in the amendment application, will be conducted in compliance with Murray State University guidelines for the protection of human participants.

The updated forms and materials that have been approved for use in this research study are attached to the email containing this letter. These are the forms and materials that must be presented to the subjects. It is your responsibility to ensure that only the updated materials are used from this point forward. Use of any process or forms other than those approved by the IRB will be considered misconduct in research as stated in the MSU IRB Procedures and Guidelines section 20.3.

This amended Level 1 protocol is valid until 2/15/2018.

If data collection and analysis extends beyond this time period, the research project must be reviewed as a continuation project by the IRB prior to the end of the approval period, 2/15/2018. You must reapply for IRB approval by submitting a Project Update and Closure form (available at murraystate.edu/irb). You must allow ample time for IRB processing and decision prior to your expiration date, or your research must stop until such time that IRB approval is received. If the research project is completed by the end of the approval period, then a Project Update and Closure form must be submitted for IRB review so that your protocol may be closed. It is your responsibility to submit the appropriate paperwork in a timely manner.

You may begin data collection using the approved changes.

Opportunity *afforded*

murraystate.edu

Equal education and employment opportunities M/F/D, AA employer. Murray State University supports a clean and healthy campus. Please refrain from personal tobacco use.
