# Supplementary material for: Exploring the stability of the gender gap in faculty perceptions of gender climate at a rural regional university
Source: PLoS One. 2024 Apr 2;19(4):e0301285. doi: 10.1371/journal.pone.0301285 (PMC10986963; doi:10.1371/journal.pone.0301285)
Supplement: S3 File — (PDF) [file pone.0301285.s004.pdf]

MURRAY STATE UNIVERSITY

Institutional Review Board

328 Wells Hall
Murray, KY 42071-3318
270-809-2916● msu.irb@murraystate.edu

TO: Maeve McCarthy, NSF ADVANCE Grant

FROM: Jonathan Baskin, IRB Coordinator JB

DATE: 11/19/2021

RE: Human Subjects Protocol I.D. – IRB # 20-001

The IRB has completed its review of your Level 1 (**Exempt – 45 CFR 46.104(d)(2) - Educational Tests, Surveys, Interviews, or Observation of Public Behavior**) protocol entitled *Leveling the Playing Field: Strategic Equity Initiatives at Murray State University*. After review and consideration, the IRB has determined that the research, as described in the protocol form, will be conducted in compliance with Murray State University guidelines for the protection of human participants.

The forms and materials that have been approved for use in this research study are attached to the email containing this letter. These are the forms and materials that must be presented to the subjects. Use of any process or forms other than those approved by the IRB will be considered misconduct in research as stated in the MSU IRB Procedures and Guidelines section 20.3.

Your stated data collection period is from 11/19/2021 to 10/31/2022.

If data collection extends beyond this period, please submit an Amendment to an Approved Protocol form detailing the new data collection period and the reason for the change.

This Level 1 determination is valid until 11/18/2022.

If data collection and analysis extends beyond this date, the research project must be reviewed as a continuation project by the IRB prior to the end of the approval period, 11/18/2022. You must reapply for IRB approval by submitting a Project Update and Closure form (available at murraystate.edu/irb). You must allow ample time for IRB processing and decision prior to your expiration date, or your research must stop until such time that IRB approval is received. If the research project is completed by the end of the approval period, then a Project Update and Closure form must be submitted for IRB review so that your protocol may be closed. It is your responsibility to submit the appropriate paperwork in a timely manner.

The protocol is approved. You may begin data collection now.

Opportunity *afforded*

murraystate.edu

Equal education and employment opportunities M/F/D, AA employer. Murray State University supports a clean and healthy campus. Please refrain from personal tobacco use.
